# Prevention and Treatment of Alzheimer's Disease Via the Regulation of the Gut Microbiota With Traditional Chinese Medicine

**DOI:** 10.1111/cns.70101

**Published:** 2024-11-07

**Authors:** Jinyao Long, Jiani Zhang, Xin Zeng, Min Wang, Ningqun Wang

**Affiliations:** ^1^ Department of Neurology Xuanwu Hospital, Capital Medical University Beijing China; ^2^ School of Life Sciences Beijing University of Chinese Medicine Beijing China; ^3^ Dongfang Hospital Beijing University of Chinese Medicine Beijing China

**Keywords:** Alzheimer's disease, inflammation, microbiota–gut–brain axis, traditional Chinese medicine

## Abstract

Alzheimer's disease (AD) is caused by a variety of factors, and one of the most important factors is gut microbiota dysbiosis. An imbalance in the gut mincrobiota have been shown to change the concentrations of lipopolysaccharide and short‐chain fatty acids. These microorganisms synthesize substances that can influence the levels of a variety of metabolites and cause multiple diseases through the immune response, fatty acid metabolism, and amino acid metabolism pathways. Furthermore, these metabolic changes promote the formation of β‐amyloid plaques and neurofibrillary tangles. Thus, the microbiota–gut–brain axis plays an important role in AD development. In addition to traditional therapeutic drugs such as donepezil and memantine, traditional Chinese medicines (TCMs) have also showed to significantly decrease the severity of AD symptoms and suppress the underlying related mechanisms. We searched for studies on the effects of different herbal monomers, single herbs, and polyherbal formulas on the gut microbiota of AD patients and identified the relevant pathways through which the gut microbiota affected AD. We conclude that improvements in the gut microbiota not only decrease the occurrence of inflammatory reactions but also reduce the deposition of central pathological products. Herbal monomers have a stronger effect on improving of central pathology. Polyherbal formulas have the most extensive effect on the gut microbiota in patients with AD. Among the effects of formulas, the anti‐inflammatory effect is the most essential and is also the main concern regarding the use of TCMs in treating AD from the viewpoint of the gut microbiota. We hope that this review will be helpful for providing new ideas for the clinical application of TCMs in the treatment of AD.

## Graphical abstract

The microbiota–gut–brain axis plays an important role in the development of Alzheimer's disease (AD). In terms of treatment, it has been proven that traditional Chinese medicine are effective in AD by regulating the gut microbiota. And then gut microbiota and its metabolism may inhibit inflammatory response through tumor necrosis factor‐α (TNF‐α), interleukin (IL), the nuclear factor kappa‐B pathway, and superoxide dismutase metabolism. The sedimentation of β‐amyloid (Aβ) and learning and memory function of AD are finally influenced. It indicates that choosing appropriate Chinese medicine as the complementary treatment may be a better way to achieve satisfactory clinical efficacy in AD.

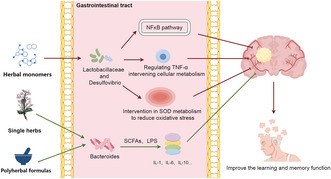

## 1 Introduction

Alzheimer's disease (AD) is a degenerative disease of the central nervous system [1]. The typical histopathological changes in AD include widespread abnormal deposition of soluble proteins, such as amyloid‐β (Aβ) and phosphorylated tau (p‐tau). These depositions cause abnormal neuronal function and glial cell proliferation [2]. The pathogenesis of AD generally involves Aβ plaque‐related neurodegeneration [3], neurofibrillary tangle formation [4], synaptic dysfunction, neurotransmitter imbalance [5], neuroinflammation [6], gut microbiota changes [7], genetic mutations [8], oxidative stress [9], autophagy [10], insulin resistance [11], etc. In recent years, the discovery of the microbiota–gut–brain axis (MGBA) has provided new ideas for studying the mechanism underlying AD pathogenesis [12]. Many scholars have started to look for ways to prevent and treat AD by regulating the gut microbiota [13, 14, 15, 16]. Recent studies revealed that traditional Chinese medicines (TCMs) may have a positive effect on AD by regulating the gut microbiota. In this review, we focused on the pathways by which TCMs regulate the MGBA to further explore the mechanism by which TCMs prevent and treat AD.

## 2 The Gut Microbiota and AD

In recent years, the enteric nervous system has been regarded as the “second brain” [17]. As humans mature and age, there is constant turnover of the dominant microorganisms in the intestine (Table 1). Different dominant microbiota communities also affect the occurrence of different diseases [18]. The abundances of *Proteobacteria*, *Gammaproteobacteria*, and *Enterobacteriaceae* were increased in patients with mild cognitive impairment [19]. AD patients have a greater abundance of *Bacteroidetes* and lower levels of *Actinobacteria, Firmicutes*, and *Bifidobacterium* in the intestine [15, 20, 21]. Foods containing *Lactobacillus* and *Bifidobacterium* may promote improvements in cognitive function in AD patients [22]. *Bifidobacterium* is an important gut microbiota that can lower cholesterol levels. It promotes cholesterol catabolism through its ability to uncouple bile salts and upregulates the low‐density lipoprotein receptor and SREBP2 expression by utilizing the high ability of cholesterol assimilation to reduce cholesterol levels [23, 24]. The substances synthesized by these microorganisms include lipopolysaccharide (LPS) and short‐chain fatty acids (SCFAs) which directly or indirectly affect brain function [25]. For example, acetate affects microglia and decreases BBB permeability [26]. Butyrate can inhibit histone deacetylase activity and affect the expression of learning‐related genes in individuals with AD [27]. LPS not only causes the release of systemic inflammatory mediators [28] but also leads to increased amyloid fibril formation and tau deposition [29].

**TABLE 1:** Disease susceptibility of the population corresponding to the dominant microbiota in different periods.

Growth stage	The dominant gut microbiota at this stage	Related system	References
Infancy	*Enterococcaceae, Staphylococcaceae, Bacteroidetes, Prevotella Streptococcus, Ruminococcus, Veillonella, Bifidobacteriaceae, Lactobacill*	Buffering system Immune system Hematologic system	[30, 31, 32]
Adolescence	*Enterobacteriaceae, Bacteroides, Clostridiales, Lachnospiraceae, Prevotella Bifidobacterium, Parabacteroides, Alistipes, Barnesville, Oscillibacter, Faecalibacterium, Fusicatenibacter, Clostridium cluster IV, Clostridium cluster XIVa (Lachnospiraceae), Bacillus, Blautia, Romboutsia, Erysipelotrichaceae, Anaerostipes*	Immune system Nervous system	[32, 33, 34]
Adulthood	*Ruminococcaceae, Veillonella, Faecalibacterium prausnitzii, Coprococcus Eubacterium ventriosum, faecalis, Rikenellaceae, Fusobacterium nucleatum, Bifidobacterium, Escherichia coli, Klebsiella, Roseburia hominis, Coprococcus eutactus, Methanobrevibacter smithii, Anaerostipes hadrus, Megasphaera micronuciformi*	Immune system Nervous system	[35, 36]
Old age	*Bacteroidetes, Proteobacteria, Bacillus, Eubacterium limosum, Clostridium cluster XIVa, Clostridium cluster IV, Akkermansia muciniphila; Anaerotruncus colihominis, Papillibacter cinnamovorans, Bifidobacteria*	Immune system	[37]

### 2.1 Correlations of the Gut Microbiota With AD Biomarkers

**FIGURE 1:**
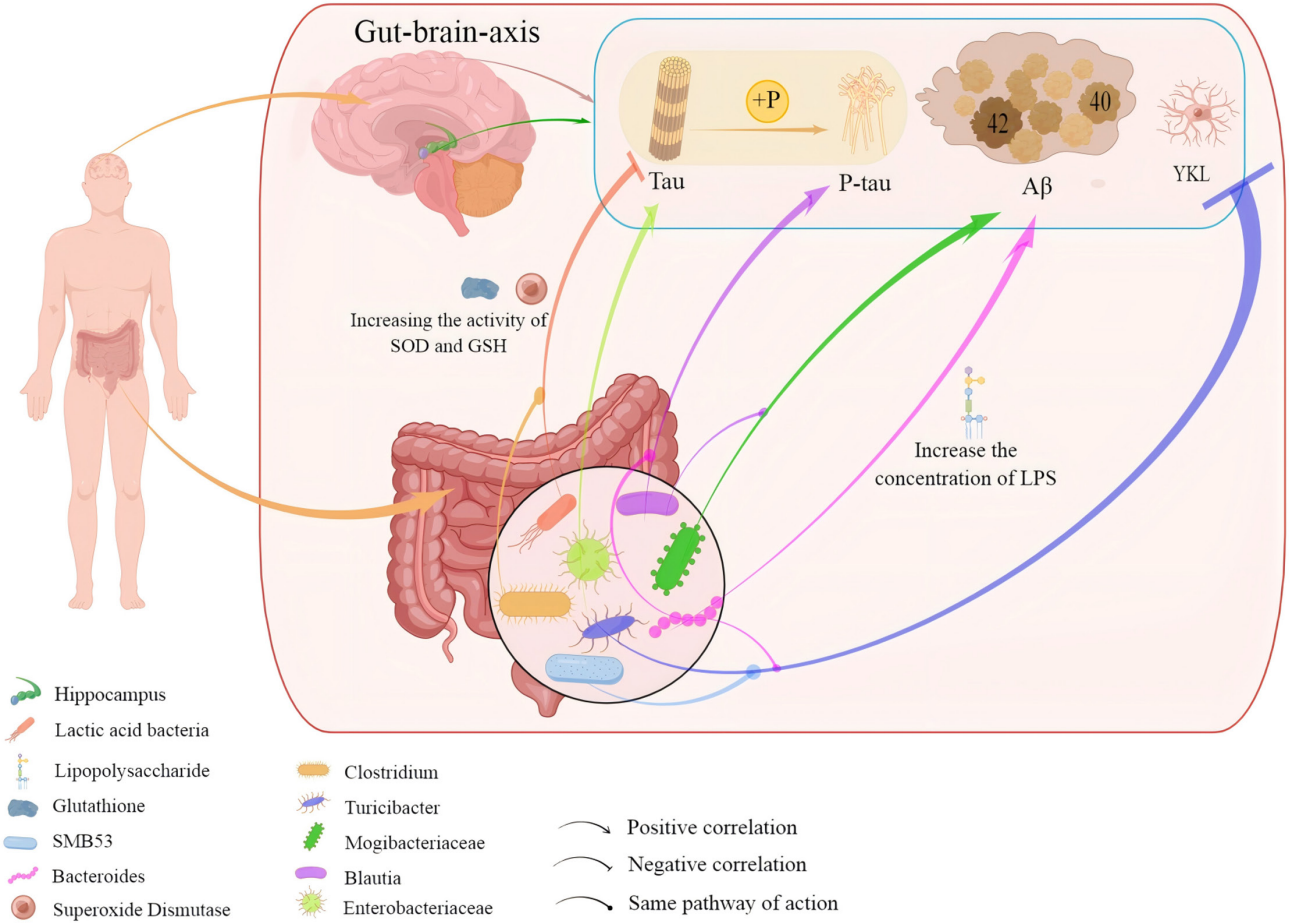
Correlations of gut microbiota with AD biomarkers.

Proteins such as Aβ, total tau, p‐tau, and chitinase‐3‐like protein 1 (YKL‐40) have been widely studied as cerebrospinal fluid (CSF) biomarkers of AD pathology [16]. A higher ratio of p‐tau to Aβ_42_ suggests a greater number of neurofibrillary tangles in the brain [38, 39]. High CSF p‐tau levels are often detected in the brains of AD patients [40]. YKL‐40 expression is significantly elevated in the cerebrospinal fluid of AD patients [41]. Changes in the concentrations of these biomarkers are usually closely related to changes in the gut microbiota. The abundances of *Mogibacteriaceae* and *Proteobacteria* were positively correlated with the ratio of Aβ_42_ to Aβ_40_ in individuals with mild cognitive impairment, and the abundance of *Enterobacteriaceae* was positively correlated with p‐tau and the ratio of p‐tau to Aβ_42_ levels [19]. The abundances of *Firmicutes* and *Actinobacteria* were lower in amyloid precursor protein / presenilin 1 (APP/PS1) mice, whereas the abundances of *Bacteroidetes* and *Tenericutes* were greater in APP/PS1 mice [42]. Consistent with the findings of Vogt's study, they also revealed that the abundances of *SMB53* (family *Clostridiaceae*), *Dialister*, *Clostridium*, and *Turicibacter* were decreased, whereas the abundances of *Blautia*, *Phascolarctobacterium*, and *Gemella* were increased in AD patients [15]. Moreover, the increase in *Bacteroides* and decrease in *Turicibacter* and *SMB53* abundances were positively correlated with YKL‐40, and the increase in *Bacteroidetes* and the decrease in *Actinobacteria* abundance led to increased deposition of Aβ and tau [15] (Figure 1).

Inflammatory factors, such as interleukin‐1 (IL‐1), interleukin‐6 (IL‐6), interleukin‐8 (IL‐8), and tumor necrosis factor‐α (TNF‐α), can also serve as potential markers for AD [43]. Peripheral IL‐1 levels are significantly greater in AD patients than in healthy individuals, and IL‐1 contributes to the deposition of Aβ and tau phosphorylation in patients with AD [44]. Increased peripheral inflammatory factor activity was positively associated with microglial and astrocyte activation in the hippocampus of 5xFAD mice [45]. Elevated IL‐6 levels are associated with early neurodegeneration in AD [46, 47]. The concentrations of IL‐8 and TNF‐α are significantly increased in the CSF of AD patients [48, 49]. TNF‐α‐targeted therapies improve cognitive function in elderly individuals [50]. In 5xFAD mice, there were decreases in the abundances of *Firmicutes*, *Bifidobacteria*, *Lactobacillus* and increases in the abundance of *Bacteroidetes* and the level of IL‐1β [51]. The abundance of *Candida tropicalis* (*C. tropicalis*) was negatively associated with IL‐8 levels, whereas the abundances of *C. tropicalis* and *Schizophyllum commune* were positively correlated with IL‐10 and TNF‐α levels in AD patients [52]. In Kim's study, compared with those in wild‐type mice, the abundances of *Burkholderiales*, *Campylobacterales*, *Barnesiella*, and *Prevotella* and the levels of TNF‐α, IL‐6, and IL‐1β were decreased in 5xFAD mice [53]. In addition, the gut microbiota‐mediated inflammatory response may represent the earliest form of neuroinflammation in AD [51].

The abundances of gut microbiota, *Turicibacter*, *SMB53*, and *Bacteroides* are negatively correlated with the levels of YKL‐40, a marker of inflammation in AD glial cells. The abundance of *Bacteroides* positively correlated with Aβ and p‐tau levels. This correlation occurs because *Bacteroides* increases the concentration of LPS, leading to increased amyloid fibril formation and tau deposition, which is consistent with *Blautia*. In addition, the abundance of *Mogibacteriaceae* is also positively correlated with Aβ levels. *Enterobacteriaceae* is positively correlated with tau levels. The abundances of *Clostridium* and *lactic acid bacteria* are related to oxidative stress and tau aggregation.

### 2.2 Effect of the Gut Microbiota on Signaling Pathways in AD

The nuclear factor kappa‐B (NF‐κB) has been implicated in AD pathology because the NF‐κB binding sites are present in the promoter regions of genes involved in Aβ production and inflammation [54, 55]. In the intestine, *Bacteroides fragilis* can promote NF‐κB transcription and activate the inflammatory response by producing LPS [56, 57]. Mitogen‐activated protein kinase (MAPK) is involved in inflammation associated with neurodegeneration and oxidative stress in individuals with AD [58]. In Li's study, the abundance of *Bacteroides acidofaciens* was significantly increased, whereas the abundances of *Clostridium methylpentosum*, *Algorimarina butyrica*, *Lawsonibacter asaccharolyticus*, *Flintibacter butyricus*, and *Alistipes finegoldii* were decreased in APP/PS1 mice. These changes contributed to lower concentration of SCFAs and promoted the production of extracellular signal‐regulated kinase [59]. Once the extracellular signal‐regulated kinase signaling is activated, it will activate CryaB and bind more strongly to Aβ, which results in the deposition of CryaB together with Aβ and these deposits cannot be degradated [60].

A decrease in glutamate levels in AD patients is associated with poor situational memory [61]. Gut microbiota‐induced glutamate metabolism is associated with cognitive function [62]. Glutamate recycling may lead to AD‐related excitotoxicity and neurodegeneration [63]. In addition, alterations in the abundances of *Bacteroides thetaiotaomicron* and *Campylobacter jejuni* are always accompanied by decreased glutamate concentrations [64, 65]. The gut microbiota‐mediated metabolism of glutamate may affect glutamate concentrations and cognitive function in AD patients [63].

Pathologically, high cholesterol levels induce Aβ deposition and tau hyperphosphorylation in APP mice [66]. In terms of symptoms, hypercholesterolemia accelerated cognitive deficits in AD patients [67, 68]. A randomized trial clearly indicated that *Lactobacillus* and *Bifidobacterium* could reduce serum triglyceride levels and have a positive effect on cognitive function in AD patients [22]. After eating food contained *Lactobacillus acidophilus*, *Lactobacillus casei*, *Bifidobacterium bifidum*, and *Lactobacillus fermentum* for approximately 12 weeks, patients had significantly lower triglyceride levels and had improved MMSE scores (27.90% ± 8.07) [69]. The improvement in the MMSE score may be due to the mechanism by which lower triglyceride levels decreased the interaction between β‐amyloid precursor protein cleavage enzyme‐1 and amyloid precursor protein [22, 70, 71].

## 3 Effect of TCMs on the Gut Microbiota in AD

**FIGURE 2:**
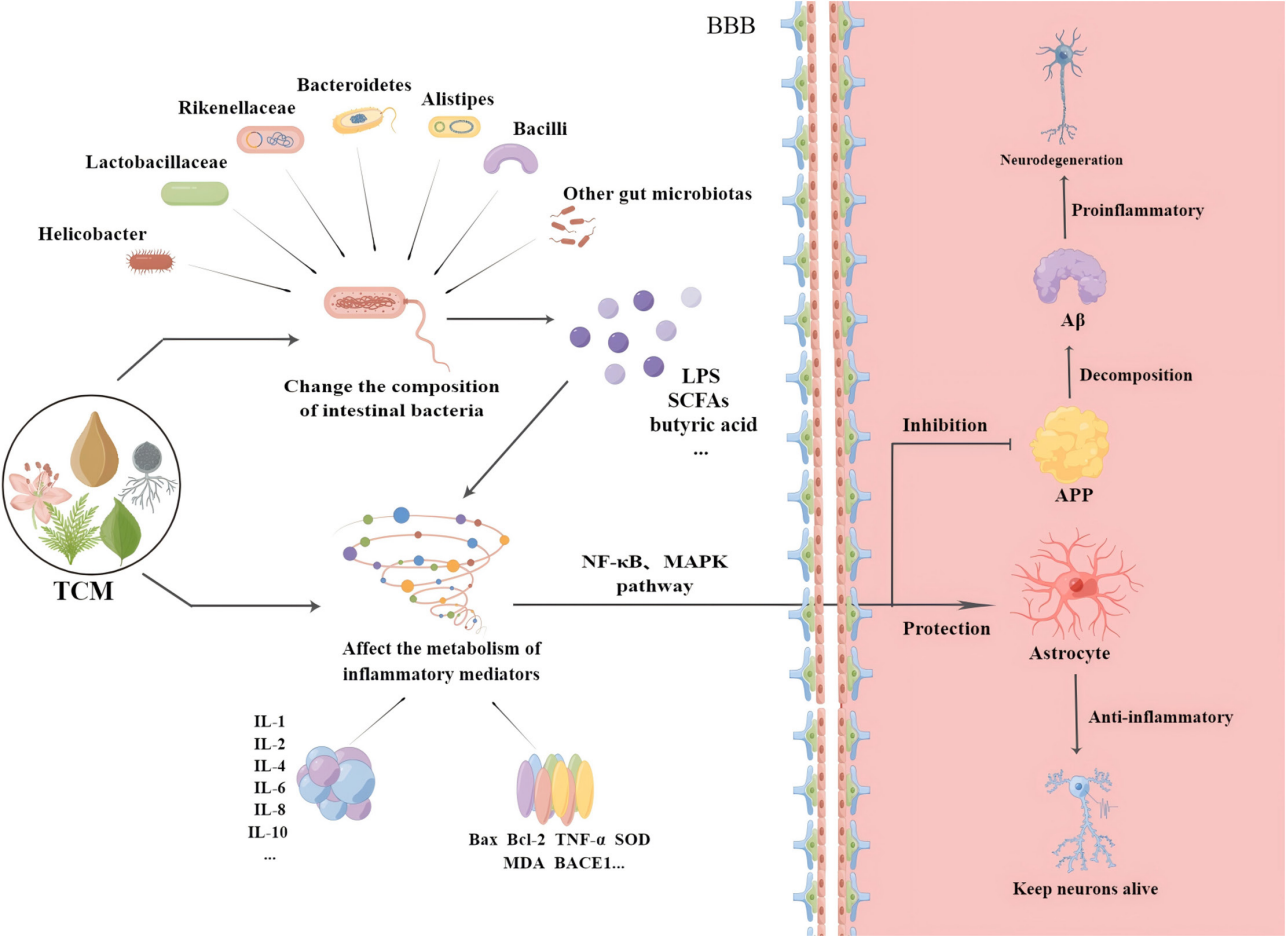
The effects of TCMs on the gut–brain–axis of AD.

TCMs have positive effects on AD, such as decreasing Aβ aggregation [72], inhibiting tau hyperphosphorylation [69], preventing neuroinflammation and apoptosis [72, 73], and promoting neuronal proliferation [74]. TCMs can decrease the levels of pathological products and improve cognitive function in AD by regulating the gut microbiota [75]. From the peripheral to the central nervous system, the gut microbiota and its metabolites play significant anti‐inflammatory and protective roles in AD [76]. In the periphery, TCMs act on inflammatory mediators through the gut microbiota and affect several inflammatory pathways. Downstream molecules of the inflammatory pathway play a role in protecting glial cells and inhibiting APP generation in the central nervous system (Figure 2).

TCMs affect inflammatory factors and the NF‐κB or MARK pathway by regulating the gut microbiota, thus interfering with the production and activation of astrocytes and APP. An increase in APP production causes neuronal degeneration in AD.

### 3.1 Herbal Monomers

#### 3.1.1 Isoorientin

Isoorientin, a natural C‐glucosyl flavonoid from Polygonum orientale, is a highly selective inhibitor of glycogen synthase kinase‐3β (GSK‐3β) [77]. Chronic administration of isoorientin to APP/PS1 mice significantly reduced GSK‐3β hyperactivation, tau protein hyperphosphorylation, and Aβ deposition in AD, and led to reverse prolonged synaptic activation and spatial memory impairment [78]. Isoorientin reduced the abundances of *Alteromonas*, *Campylobacterales*, and *uncultured Bacteroidales bacterium* and significantly reduced Aβ_42_ deposition and p‐tau deposition in the hippocampus and cortex in APP/PS1 mice [79]. Isoorientin partially reversed the decreased abundances of *Muribaculaceae* and *Prevotellaceae* and the increased abundance of *Erysipelotrichaceae* in scopolamine‐induced AD mice [80]. These microorganisms were related to inflammatory factors and pathways, including the TNF‐α, IL‐4, IL‐10, GSK‐3β, MAPK, and NF‐κB pathways [79, 80].

#### 3.1.2 Curcumin

Curcumin is isolated from *Curcuma longa*. It has antioxidant, anti‐inflammatory, and neuroprotective effects and has been proven to be a promising anti‐AD compound [81]. Curcumin was shown to alter the abundances of the families *Lactobacillaceae* and *Rikenellaceae* and the genera *Prevotella*, *Bacteroides*, and *Parabacteroides* in the intestines of APP/PS1 mice [82]. *Bacteroides* species increased the permeability of the gastrointestinal barrier. Through this process, *Bacteroides* species promoted the entry of pathogens, LPS, and certain metabolites into the bloodstream [83]. This resulted in a reduction in the number of amyloid plaques in the hippocampus and an improvement in spatial learning and memory in APP/PS1 mice [83]. Curcumin promoted neuroinflammation via the activation of the NF‐κB signaling pathway and this pathway may be a potential mechanism by which curcumin blocks the occurrence of AD [84].

#### 3.1.3 Ginsenoside

Ginsenoside is one of the active components of *Panax ginseng*, and has been extensively used to treat dementia [85]. From the pharmacological perspective, ginsenoside significantly inhibits neurotoxicity, regulates nerve growth factor levels, and promotes neurological recovery [86]. In a study of AD tree shrews, the abundance of *Lactobacillus salivarius* and the ratio of *Firmicutes/Bacteroides* in the ginsenoside treatment group were markedly greater than those in the AD group [87]. Moreover, compared with those in the AD tree shrew model group, Bcl‐2‐associated X (Bax), beta‐secretase 1, Aβ_1‐42_, and p‐tau levels were significantly lower and the learning and memory abilities were improved in the ginsenoside treatment group [87, 88].

#### 3.1.4 Resveratrol

Resveratrol is a neuroprotective polyphenolic compound. It is one of the main active ingredients of *Reynoutria japonica*. It has antioxidant, anti‐acetylcholinesterase, and antiapoptotic effects [89]. Resveratrol reduces the Aβ concentration by reducing the cleavage of APP and inhibiting amyloidogenic pathways, reversing the learning and memory impairments [90]. Besides, resveratrol supplementation facilitates the dephosphorylation of tau and inhibits its aggregation [91, 92]. When resveratrol was administered by gavage for 16 weeks in the D‐galactose (D‐gal)‐induced mice, researchers reported that resveratrol was able to regulate Aβ production associated with oxidative stress by modulating the gut microbiota, including *Lactobacillus*, *Erysipeltrichales*, and *Faecalibaculum* [93]. Resveratrol also significantly increased the abundances of *Lactobacillus*, *Bifidobacterium*, *Allobaculum*, *Coriobacteriaceae_UCG‐002*, *Alloprevotella*, *Candidatus_Saccharimonas*, *Alistipes*, and *Parasutterella*, and reduced the abundances of *Rikenella*, A*naerotruncus*, *Colidextribacter*, and *Helicobacter* [91]. They decreased reactive oxygen species levels and insulin resistance and increased the synthesis of superoxide dismutase (SOD) to ameliorate cognitive decline and neuroinflammation [91, 93].

#### 3.1.5 *Cistanche deserticola* Polysaccharide

*Cistanche deserticola* polysaccharide (CDPS) is one compound of *Cistanche deserticola* that exerts anti‐inflammatory, antioxidant, antiaging, neuroprotective, and immunomodulatory effects [94]. CDPS also plays a significant role in improving the gut microbiota of AD. D‐gal‐induced aging mice were given CDPS by gavage, and the abundances of *Bacillus*, *unidentified Actinobacteria* and *Fusobacteria* were significantly decreased and the abundances of *Methanobacteria*, *Deltaproteobacteria*, *Anaerolineae*, and *Erysipelotrichia* were increased [95]. The levels of IL‐2 and TNF‐α were decreased and the IL‐4 and IL‐10 levels were significantly increased, moreover, the learning and memory functions were improved [95]. However, when triple antibiotics and cyclophosphamide were applied to sterilize the gut microbiota, the brain, and serum levels of proinflammatory cytokines, including TNF‐α, IL‐2, SOD, and malondialdehyde, were significantly increased [95].

#### 3.1.6 Ginkgolide B

Ginkgolide B is a diterpene lactone isolated from *Ginkgo biloba*. By regulating Aβ‐induced endoplasmic reticulum stress, it protects astrocytes and alleviates cognitive impairment in AD [96]. In addition, ginkgolide B treatment significantly reduced the levels of Bax and increased the level of Bcl‐2 in D‐gal and aluminum chloride (AlCl3/D‐gal)‐induced mice by decreasing the abundances of *Bacteroidales, Muribaculaceae*, and *Alloprevotella*, and increasing the abundance of *Lactobacillus* [97]. In this study, the mice were administered ginkgolide B by gavage for 2 weeks, the ability of learning and memory were significantly improved [97].

#### 3.1.7 Xanthoceraside

Xanthoceraside is a novel triterpenoid saponin obtained from the husks of *Xanthoceras sorbifolia*, which has been shown to ameliorate encephaledema and neuroinflammatory diseases [98]. Xanthoceraside also attenuates Aβ_1‐42_‐induced memory impairment by suppressing the neuroinflammatory response [99]. AD rats injected with Aβ_1‐42_ were administered xanthoceraside by gavage, and the abundances of *Methanomassiliicoccus*, *Azoarcus*, *Phycisphaera*, *Clostridium IV*, *Enterorhabdus*, *Coriobacterium*, *Acetobacteroides*, and *Alloprevotella* were regulated to decrease SCFA levels and improve the memory and cognitive impairment in AD rats [100].

At present, herbal monomers are a key type of TCMs used to treat diseases. This article searches herbal monomers with therapeutic effects on AD and list their pathway and targets on AD in the following (Table 2).

**TABLE 2:** Potential mechanisms of herbal monomers in the modulation of the gut microbiota for the treatment of AD.

Herbal monomer	Pharmacological action	Mainly affected microbiota or microbial metabolites	Related targets and pathways	Effects on AD	References
Isoorientin	A highly selective inhibitor of GSK‐3β; reverses the prolonged activation of synapses	Reduces the abundances of *Alteromonas*, *Campylobacterales*, and *uncultured Bacteroidales bacterium;* reduces LPS levels in the brain and serum	Suppresses core components of the MAPK and NFκB pathway; reduces the concentration of TNF‐α, IL‐4, IL‐10, and GSK‐3β	Reduces Aβ and brain tau protein levels and phosphorylates p65; reverses prolonged synaptic activation and spatial memory impairment in APP/PS1 mice	[78, 79]
Curcumin	Antioxidant, anti‐inflammatory, and neuroprotective effects	Increases the abundances of the families *Lactobacillaceae* and *Rikenellaceae* and the genera *Prevotella*, *Bacteroides*, and *Parabacteroides*	Activates the NF‐κB signaling pathway	Reduces neuroinflammation and Aβ deposition; improves spatial learning and memory ability in APP/PS1 mice	[83, 84]
Ginsenosides	Inhibits inward Ca^2+^ flow and neurotoxicity; anti‐inflammatory, Antioxidant, anti‐apoptotic effects; protects mitochondria; regulates nerve growth factor expression and promotes nerve recovery	Increases the abundance of *Lactobacillus salivarius*; decreases the abundance of *Bacteroidetes*	Reduces the concentrations of Bax and beta‐secretase 1	Reduces Aβ_1‐42_ and p‐tau deposition; improves learning and memory impairment in AD tree shrews	[86, 87, 88]
Resveratrol	Inhibits acetylcholinesterase activity; antioxidant, anti‐apoptotic effects; stimulates cell autophagy; reduces neuroinflammation	Reduces the abundances of *Alistipes* and *Helicobacter*; increases the abundance of *Desulfovibrio*	Reduces reactive oxygen species production and increases SOD synthesis	Reduces the cleavage of APP; inhibits Aβ production; ameliorates cognitive decline in D‐gal mice	[89, 90, 91, 92, 93]
CDPS	Anti‐inflammatory, antioxidant, neuroprotective, and immunomodulatory effects	Decreases the abundances of Bacillus and unidentified Actinobacteria; increases the abundances of Fusobacteria, Methanobacteria, Spirochaetia, Deltaproteobacteria, Nitrososphaeria, Anaerolineae, and Erysipelotrichia	Decreases TNF‐α, IL‐2, SOD and malondialdehyde concentrations; increases IL‐4 and IL‐10 concentrations	Reduces Aβ accumulation and p‐tau aggregation; improves learning and memory functions in D‐gal mice.	[94, 95]
Ginkgolide B	Activates the MAPK pathway; regulates endoplasmic reticulum stress and oxidative stress	Decreases the abundances of *Bacteroidales*, *Muribaculaceae*, and *Alloprevotell*a; elevates the abundance of *Lactobacillus*	Decreases the levels of Bax; increases the level of Bcl‐2	Alleviates neurodegeneration; decreases amyloid deposition; protects Nissl bodies; improves the learning and memory in D‐gal mice	[96, 97]
Xanthoceraside	Ameliorates cerebral edema and neuroinflammation	Increases the abundances of Methanomassiliicoccus, Azoarcus, Phycisphaera, Acetobacteroides, and Alloprevotella; reduces the abundances of Clostridium IV, Enterorhabdus, Coriobacterium, Corynebacterium, Desulfovibrio, and Defluviitale	Affects amino acid metabolic pathways	Promotes microglial maturation reduces memory and cognitive impairment in AD rat injected with Aβ_1‐42_	[98, 99, 100]

### 3.2 Single Herbs

#### 3.2.1 Forsythia suspensa

*Forsythia suspensa* exerts positive effects in AD patients [101]. However, the specific mechanism is not very clear. After extracts of *Forsythia suspensa* were administered by gavage at 200 mg/kg/day in Aβ‐induced AD rats for 50 days, the abundances of *Erysipelotrichales*, *Clostridiales*, *Coriobacteriales*, *Desulfovibrionales Bacteroidales*, *Lactobacillales*, *and Bacillales* were increased in the *Forsythia suspensa* group [102]. The *Forsythia suspensa* significantly inhibited the inflammatory response and tau deposition in the hippocampus and it also improved the memory function in AD rats [102]. There are several components of *Forsythia suspensa* that have been identified. Among these compounds, flavonoids and phenyl ethanol glycosides have neuroprotective effects and have significant effects on AD [103], whereas phenyl ethanol glycosides inhibit the LPS‐induced activation of BV2 microglia through the NF‐κB, MAPK, and other pathways [104]. These effects are accompanied by significant anti‐neuroinflammatory, antioxidant, antibacterial, and antiviral effects [105].

#### 3.2.2 Rheum officinale

*Rheum officinale* has been proven to improve cognitive impairment in AD rats via several pathways, including the NF‐κB and PI3K‐AKT pathways [106]. *Rheum officinale* affected IL‐18, IL‐1, and TNF‐α concentrations by regulating the abundances of *Bacillus* and *Anthropoid* and it also ameliorated the pathological damage, such as oxidative stress and neuroinflammation in APP/PS1 mice [107]. The main active ingredients of Rheum officinale are the anthraquinone derivatives emodin, aloe‐emodin, chrysophanol, rhein, physcion, and danthron [108]. According to modern pharmacological studies, these ingredients could improve the cognitive function of AD patients by interfering with the PI3K‐AKT pathway and inflammatory factors [106]. Besides, they play roles in inhibiting microglial activation, reducing synaptic dysfunction, and decreasing tau phosphorylation [109].

#### 3.2.3 Poria cocos

Poria cocos has a good acetylcholinesterase inhibitory effect and affects AD [110]. Through antioxidant and antiapoptotic effects, Poria cocos protects neuronal cells from Aβ‐induced injury [111]. When Poria cocos extract was administered by gavage at 1.2 g/kg/day for 3 months, the abundances of *Deferribacteraceae*, *Lachnospiraceae*, and *Enterobacteriales* were decreased and cognitive impairment was alleviated in APP/PS1 mice [112]. Poria cocos directly interfered with the synthesis of Aβ plaques in the brain tissue of APP/PS1 mice by reversing the abnormal metabolism of β‐secretase and γ‐secretase [112, 113].

#### 3.2.4 Gastrodia elata

*Gastrodia elata* (GE) is a commonly used TCM for treating neurological diseases such as headache, AD, and Parkinson's disease [114]. After treatment with GE by gavage for 4 months, the abundances of probiotic bacteria, such as *Bifidobacterium*, *Akkermansia*, and *Alloprevotella* increased in ApoE mice and these changes in the gut microbiota increased the SCFAs concentration and decreased the serum corticosterone concentration [115]. By regulating brain monoamine neurotransmitter and Aβ levels, GE alleviated exploratory behavior and recognition ability in ApoE mice [115]. In the modern pharmacological studies, GE promotes synaptic regeneration and exerts neuroprotective effects by interfering with signal transduction [116]. GE can also reduce the levels of oxidative stress‐related products such as malondialdehyde and glutathione disulfide [117]. GE also has an important effect on reducing neuronal loss and apoptosis in AD, and this process is achieved mainly through activation of the PI3K/AKT/mTOR pathway, suppression of the NLRP3/NF‐κB pathway and the release of the apoptotic proteins Bcl‐2 and Bax [118]. GE ameliorates cognitive impairment by inhibiting oxidative stress and anti‐inflammatory pathways [119]. Gastrodin and gastrodigenin are the two active components of GE, and they can also reduce mitochondrial dysfunction caused by oxidative damage [120].

The purification of herbal monomers and the development of formulas are based on an understanding in the efficacy of single herbs. The following table lists the roles and related pathways of single drugs in the gut microbiota of AD patients (Table 3).

**TABLE 3:** Effects of single herbs on the gut microbiota and AD.

Single herb	Pharmacological action	Mainly affected microbiota or microbial metabolites	Related targets and pathways	Effects on AD	Reference
*Forsythia suspensa*	Inhibits activation of BV2 microglia; anti‐neuroinflammatory, antioxidant, antibacterial, antiviral and neuroprotective effects	Maintains the activity of Erysipelotrichales, Clostridiales, Coriobacteriales, Desulfovibrionales Bacteroidales, Lactobacillales, and Bacillales; increases the butyric acid concentration in serum	Enhances intestinal barrier function and mucosal immunity; reduces the absorption of bacteria	Attenuates the inflammatory response in the central nervous system; improves memory function in AD rats injected with Aβ_1‐42_	[102, 103, 121]
*Rheum officinale*	Affects the PI3K‐AKT pathway and TNF‐ α, IL‐6, and SOD levels; inhibits the activation of microglia; reduces synaptic dysfunction	Regulates the abundances of *Bacillus* species and *anthropoid bacteria*	Influences the concentrations of IL‐8, IL‐1, and TNF‐α	Reduces tau phosphorylation to improve learning and memory function in AD; improves AD‐related oxidative stress and neuroinflammation in APP/PS1 mice	[106, 107, 109]
Poria cocos	Reverses the abnormal metabolism of β‐secretase and γ‐secretase	Decreases the abundances of *Deferribacteraceae*, *Lachnospiraceae*, and *Enterobacteriales*; increases the abundance of *Lactobacillus*	Reduces neurotoxic amino acid levels and the production of TNF‐ α and IL‐6; regulates bile acid metabolism	Affects the synthesis of Aβ plaques in the brain tissues of APP/PS1 mice; alleviates cognitive impairment in APP/PS1 mice	[112]
GE	Reduces the levels of malondialdehyde and SOD; promotes synapse regeneration; neuroprotective effects; reduces neuronal loss and apoptosis; reduces mitochondrial dysfunction	Increases the abundances of Bifidobacterium, Akkermansia, Alloprevotella	Increases the concentration of SCFAs; reduces serum corticosterone levels	Regulates brain monoamine neurotransmitter and Aβ_1–42_ levels; alleviates exploratory behavior and recognition ability in ApoE mice	[115, 116, 117, 118, 119, 120]

### 3.3 Polyherbal Formulas

#### 3.3.1 Huang Lian Jie Du Decoction

Huang Lian Jie Du (HLJD) contains four herbs, including Coptis chinensis, Scutellaria baicalensis, Phellodendron amurense, and Gardenia jasminoides. HLJD is a representative decoction for heat‐clearing and detoxicating, and has been widely used to treat neurological disorders [122, 123] HLJD can improve cognitive function, reduce the plaque burden, decrease inflammation and oxidative stress [124, 125, 126]. Through these effects, HLJD can alleviate learning and memory deficits in AD mice [127]. When HLJD was administered by gavage for 4 months, the gut microbiota was regulated especially by increasing the abundances of *Prevotellaceae*, *Lactobacillaceae*, *Peptococcaceae*, *Alcaligenaceae*, and *Helicobacteraceae* and reducing the abundances of *BacteroidalesS24‐7_group*, *Lachnospiraceae*, and *Porphyromonadaceae*, which ameliorated the memory and spatial learning deficits in APP/PS1 mice [76]. Coptis chinensis is the monarch herb of HLJD. By increasing the abundances of *Firmicutes*, *Bacteroidetes*, and *Lachnospiraceae* and decreasing the abundance of *Verrucomicrobia, Coptis chinensis* significantly promotes the production of SCFAs [128] and its therapeutic effects can also be achieved via SCFAs [129].

#### 3.3.2 Liuwei Dihuang Decoction

Liuwei Dihuang (LWDH) is a classical formula that is widely used to treat AD [130]. It contains six herbs, including Rehmannia glutinosa, Dioscorea oppositifolia, Cornus officinalis, Alisma plantago‐aquatica, Poria cocos, and Paeonia × suffruticosa [131]. According to modern pharmacological studies, LWDH can regulate the neuroimmune network by reducing the levels of interleukins, colony‐stimulating factors, TNF‐α and chemotactic factors [132]. Moreover, it can improve cognitive ability and neuronal synaptic function in animal models of aging or AD [133]. LWDH may exert a neuroprotective effect on AD by regulating the gut microbiota. When senescence‐accelerated mouse prone 8 (SAMP8) mice were given LWDH by gavage for 90 days, the abundances of *Bacteroidales*, *Clostridiales*, and *Desulfovibrionales* were altered and spatial learning and object recognition memory were improved in SAMP8 mice [134]. As a monarch medicine of LWDH, Rehmannia glutinosa can improve cognitive function by decreasing the levels of IL‐1β and TNF‐α [135]. Cornus officinalis is one of the minister herbs in LWDH, and it can increase the levels of SCFAs and regulate the levels of IL‐1β and TNF‐α by decreasing the abundances of *Bacteroidetes* and *Proteobacteria* [136]. The interaction between *Rehmannia glutinosa* and *Cornus officinalis* makes MGBA play an important role in AD [137].

#### 3.3.3 Qisheng Wan Formula

Qisheng Wan (QSW) has been widely used to treat patients with amnesia or dementia [138]. It consists of seven substances, including Poria cocos, Neolitsea cassia, Panax ginseng, Polygala tenuifolia, Asparagus cochinchinensis, Acorus gramineus, and Lycium chinense. The abundances of *Akkermansia*, *Lactobacillus*, and *Bifidobacterium* were increased and the abundance of *Lactobacillus* was reduced in Aβ_1‐42_‐induced AD rats after being administrated QSW by gavage for 4 weeks [139]. Changes in these microorganisms were accompanied by the increased levels of NF‐κB, IL‐6, and TNF‐α, and improved the spatial learning and memory abilities of AD rats [139].

#### 3.3.4 Qi‐Fu‐Yin Formula

Qi‐Fu‐Yin (QFY) is a typical prescription used to treat dementia [140, 141], and it is composed mainly of seven herbs, including Panax ginseng, Rehmannia glutinosa, Angelica sinensis, Atractylodes macrocephala, Polygala tenuifolia, Glycyrrhiza uralensis, and Ziziphus jujuba. QFY can reduce the ratio of Aβ_1‐42_ to Aβ_1‐40_ and the concentrations of IL‐1β and TNF‐α [142]. The anti‐inflammatory effect inhibited the excessive activation of microglia and increased synaptic plasticity in the hippocampus [143]. QFY improves object recognition ability, passive avoidance responses, and spatial learning and memory abilities in AD animals [144, 145]. When QFY was administered by gavage for 3 months, the abundances of *Bacteroides*, *Bacteroidaceae*, and *Rikenellaceae* were significantly reduced and the abundance of *Erysipelotrichaceae* was increased in APP/PS1 mice [146]. QFY also inhibits the activation of aspartyl‐tRNA synthesis and improves spatial learning and memory abilities and promotes the recovery of motor coordination in AD mice [146].

#### 3.3.5 Modified Huang‐Lian‐Jie‐Du Decoction

Modified Huang‐Lian‐Jie‐Du (M‐HLJD) is a form of HLJD without *Scutellaria baicalensis* but with *Salvia miltiorrhiza*, *Curcuma longa*, and *Acorus gramineus*. Many studies have shown its well‐improvement effects in AD [147]. M‐HLJD relieves neuroinflammation and decreases Aβ deposition and tau phosphorylation in AD [148]. When M‐HLJD was administered intragastrically for 3 weeks, glutamate energy transmission and adenosine‐related signaling pathways were regulated through a process mediated by the N‐methyl‐D‐aspartate receptor in C57BL/6 mice injected with Aβ_1‐42_ [149]. In addition, M‐HLJD significantly reduced the abundances of *Bacteroides*, *Paraacteroides*, *Mycoplasmataceae*, and *Firmicutes* and increased the abundances of *Rikenella* and *Oscillospira*. By altering the abundance of the gut microorganisms, M‐HLJD inhibited adenosine signaling, which further ameliorates synaptic plasticity and learning and memory deficits in AD models [149]. *Salvia miltiorhiza* plays an important role in anti‐AD effects by binding to Aβ and inhibiting its aggregation [150]. Tanshinone IIA is a compound of *Salvia miltiorhiza* that upregulates the expression of insulin‐degrading enzymes and reduces the deposition of Aβ in the brain. By reducing oxidative stress and inhibiting neuronal apoptosis, tanshinone IIA increased cholinergic system activity and led to improvements in learning ability in APP/PS1 mice [151]. Thus, tanshinone IIA is one such agent and has a key effect on the treatment of AD.

#### 3.3.6 Jiedu‐Yizhi Formula

Jiedu‐Yizhi (JDYZ) has been proven to alleviate the cognitive impairment in AD [152]. JDYZ is composed of *Coptis chinensis*, wine‐treated *Rheum officinale*, *Conioselinum anthriscoides*, *Kummerowia striata*, *Persicaria perfoliata*, *Cornus officinalis*, and *Alpinia oxyphylla*. JDYZ can reduce the levels of the anti‐inflammatory factors, increased the number of neurons and decreased the deposition of Aβ in the hippocampus of Aβ_25‐35_‐induced rats [153]. The anti‐inflammatory effects of JDYZ in AD may be related to its regulation of the gut microbiota [154]. When JDYZ was administered intragastrically for 8 weeks, the abundances of *Firmicutes*, *Campilobacterota* and *Desulfobacterota* were reduced while the abundances of *Bacteroidoota* and *Actinobacterota* were significantly increased in AD rats injected with Aβ_25‐35_ [155]. These microorganisms maintained the integrity of the intestinal mucosal barrier, which was achieved mainly through reducing the expressions of Caspase‐1 and Caspase‐11, apoptosis‐related proteins, and the inflammatory pathways [155]. By intervening aforementioned factors, JDYZ significantly improved the learning and memory abilities of AD model rats.

#### 3.3.7 Ling Gui Zhu Gan Decoction

Ling Gui Zhu Gan (LGZG) contains four herbs, including Poria cocos, Cinnamomum verum, Atractylodes macrocephala, and Glycyrrhiza glabra, and has anti‐inflammatory and antioxidative stress effects [156]. It exerts anti‐inflammatory effects by reducing the generation of TNF‐α, IL‐1β, IL‐6, malondialdehyde, and ROS in Aβ_25‐35_‐induced cells [157]. Besides, LGZG can inhibit the overexpression of inflammatory factors and activate microglia to protect cells against Aβ_25‐35_‐induced injury [158]. Numerous studies have shown that LGZG can exert its effects by regulating the gut microbiota and its metabolites [159, 160, 161]. When the LGZG decoction was administered by gavage for 9 weeks, the abundances of *Bifidobacterium*, *Paraprevotella*, and *Dubosiella* were increased, along with increased SCFAs and anti‐inflammatory effects mediated by the MAPK signaling pathway in AlCl3/D‐gal‐induced mice [162].

In the perspective of TCM treatment for AD, the treatment direction can be varied at different stages. Sometimes it needs to supplement deficiency, and sometimes it needs to relieve excess. The formulas for supplementing deficiency and relieving excess also have different effects on the changes in the gut microbiota of AD. The following table lists the different formulas regulating the gut microbiota in AD (Table 4).

**TABLE 4:** Effects of polyherbal formulas on the gut microbiota and Alzheimer's disease (AD).

Polyherbal formula	Pharmacological action	Mainly affected microbiota or microbial metabolites	Related targets and pathways	Effects on AD	Reference
HLJD	Improves cognitive function; reduces the plaque burden and oxidative stress; alters lipid metabolism	Decreases the abundances of *Prevotelaceae* and *Lachnospiracea*; increases the concentrations of SCFAs and the abundances of *Bacteroides* and *Lactobacillus*; maintains intestinal homeostasis	Reduces the ratio of IL‐1β/IL‐10; alters bile acid metabolism	Improves cognitive impairment in APP/PS1 mice	[76, 122, 123, 124]
LWDH	Regulates the neural immune network; reduces the concentrations of IL, colony‐stimulating factor, TNF, and chemotactic factors	Changes the abundances of *Bacteroidales*, *Clostridiales*, *Desulfovibrionales* and other bacteria	Increases the level of serotonin	Improves the spatial learning, memory capacity and object recognition memory capacity of SAMP8 mice	[133, 134, 163, 164]
QSW	Inhibits the activity of cholinesterase in the brain; protects the organization of the brain after ischemia and hypoxia; improves immune function	Increases the abundances of *Akkermansia*, *Lactobacillus*, and *Bifidobacterium*; reduces the abundances of *Alistipes* and *Lachnospiraceae*	Alters the levels of inflammatory factors such as NF‐κB, IL‐6, and TNF‐α	Improves the spatial learning and memory abilities of AD rats injected with Aβ_1‐42_	[138, 139]
QFY	Inhibits the excessive activation of microglia; increases synaptic plasticity in the hippocampus	Reduces the abundances of Bacteroidetes, Rikenellaceae, and Firmicutes	Alters carbohydrate active enzyme and cellular metabolic processes, such as inhibiting the activation of aspartyl‐tRNA synthesis	Improves the spatial learning, memory and the motor coordination abilities of APP/PS1 mice	[144, 145, 146, 165]
M‐HLJD	Regulates N‐methyl‐D‐aspartate receptor‐mediated glutamate transmission; neuroprotective effects	Reduces the abundances of *Bacteroides*, *Paraacteroides*, and *Mycoplasmataceae* as well as *Firmicutes*; increases the abundances of *Rikenella* and *Oscillospira*	Mediates signal transduction via the adenosine pathway	Alleviates the dysfunction of synaptic plasticity and learning and memory deficits in APP/PS1 mice	[81, 148, 149, 151]
JDYZ	Inhibits the occurrence of inflammation; increases the number of neurons in the hippocampus	Increases the abundances of Bacteroidota and Actinobacteriota; reduces the abundances of Firmicutes, Campilobacterota, and Desulfobacterota	Increases the concentrations of SCFAs and LPS; downregulates the expression of Caspase‐1 and Caspase‐11; inhibits pyroptosis‐related proteins and the inflammatory pathway	Decreases the deposition of Aβ; improves learning and memory function in AD model rats injected with Aβ_1‐42_	[153, 154, 155]
LGZG	Anti‐inflammatory and anti‐oxidative stress effects; reduces insulin resistance; regulates bile acids metabolism	Increased the abundances of Bifidobacterium, Paraprevotella, and Dubosiella	Enhances SCFA production; exerts anti‐inflammatory by MAPK signaling pathway	Decreases the deposition of Aβ and the production of proinflammatory cytokines; mitigates cognitive impairment in AlCl3/D‐gal mice	[156, 157, 158, 159, 160, 161, 162, 166]

## 4 Discussion and Prospects

As the number of studies on the MGBA has increased, the understanding of the pathological mechanism in AD has increased. The gut microbiota influences the initiation and progression of AD and plays an important role in the treatment of AD. Neurofibrillary tangles and Aβ plaques are significantly lower in AD model animals that receive normal intestinal colonizing bacteria than in normal germ‐free AD model animals [14]. *Bacteroidetes*, *Firmicutes*, *Bifidobacterium, and Lactobacillus* are the main gut microbiota that cause AD along with AD‐related neuroinflammation in patients [18]. Maintaining the stability of the gut microbiota is important for the prevention and treatment of AD. TCMs have good therapeutic effects in this regard. In the treatment of bacterial dysbiosis related to AD, TCMs mainly change the concentrations of SCFAs and LPS by reducing harmful microbiota and increasing beneficial bacteria. SCFAs and LPS further affect inflammatory factors and pathways associated with AD. These inflammatory factors and pathways mainly include the interleukins, Bax, Bcl‐2, and TNF‐α, and the pathways mainly include the NF‐κB pathway and bile acid metabolism. Through these processes, significant improvements in cognitive ability can be achieved in AD.

From numerous studies on the effects of herbal monomers, single herbs, and polyherbal formulas on the gut microbiota of AD, we concluded that some TCMs exert significant anti‐inflammatory effects in AD and improve the symptoms of AD. These TCMs include *Coptis chinensis*, *Cistanche deserticola* and *Poria cocos*, which are in commonly used in the clinical treatment of AD. On the basis of the effects of the monomers in gut microbiota and AD, we can see that most monomers can act on Aβ and tau in the brain. The possible mechanism may be that the molecular weight of the monomer is relatively small, which can cross the BBB easily to reduce the pathological depositions in AD. The main pathway involved is the NF‐κB pathway and many inflammatory factors. Herbal monomers mainly regulate the abundances of *Lactobacillaceae* and *Desulfovibrio* to affect the concentrations of interleukins, Bax‐1, and TNF‐α and the metabolism of SOD. These substances can help to reduce the Aβ and tau deposition in AD. Single herbs mainly regulate the gut microbiota in *Bifidobacterium* and *Bacteroides* to affect the concentrations of interleukins and TNF‐α et al. Polyherbal formulas mainly alter the levels of SCFAs and LPS in the intestine through *Bacteroidetes*, *Firmicutes*, *Bifidobacterium, and Lactobacillus*. Compared with monomers and single drugs, polyherbal formulas have more ingredients and targets, thus, polyherbal formulas regulate the gut microbiota more extensively. By regulating the levels of interleukins, such as IL‐1, IL‐6, and IL‐10, TCMs inhibit the inflammatory reactions and Aβ deposition.

Although we have tried our best to search the literatures on TCMs, this review may not include all TCMs that have already been studied in the gut microbiota. Although numerous studies have been conducted on the regulation of the gut microbiota by TCMs in the treatment of AD, the mechanism is still incompletely understood. Besides, the complexity of the gut microbiota makes it difficult to identify the corresponding intracranial targets. Meanwhile, the mechanism of AD is still unclear. All of the above factors make it hard to further explore the relationship between TCMs, the gut microbiota and AD. Moreover, current studies are mostly in the laboratory stage, and more clinical data are needed to support the conclusions of the laboratory experiments. Therefore, more studies are needed to explore the relationships among TCMs, the gut microbiota and AD, and then we need to gradually shift the focus from laboratory to clinical research.

All the plant names have been checked with http://mpns.kew.org.

## Author Contributions

**Jinyao Long, Jiani Zhang:** conceptualization and original draft preparation. **Xin Zeng, Min Wang:** writing – reviewing and editing. **Ningqun Wang:** supervision and revision of the manuscript.

## Conflicts of Interest

The authors declare no conflicts of interest.

## Data Availability

The authors have nothing to report.
